# PREVENTABILITY OF DEMENTIA IN PRIMARY CARE SETTINGS

**DOI:** 10.1093/geroni/igz038.426

**Published:** 2019-11-08

**Authors:** Anh Pham, Neil Drummond, Don Voaklander, Adrian Wagg, Cliff Lindeman

**Affiliations:** 1 University of Alberta, Edmonton, Alberta, Canada; 2 University of Alberta, Edmonton. Alberta, Alberta, Canada

## Abstract

Dementia is a long-term, chronic condition caused by a progressing physical damage in the brain. Evidence suggests that cardiovascular disease risk factors may contribute to the onset of dementia; however, the current literature on this association is inconsistent. To our knowledge, no study that has explored the occurrence of cardiovascular risk factors prior to a diagnosis of dementia using national primary care data in North America. We used electronic medical records from the Canadian Primary Care Sentinel Surveillance Network to create a Canadian cohort to conduct a retrospective analysis to (1) determine the number of incident diagnoses of dementia in primary care among community-dwelling seniors; (2) compare the risk of developing dementia in seniors (aged 65 and older) with and without modifiable cardiovascular risk factors. The cohort identified 21,628 patients who did not have a dementia diagnosis in 2008. During ten years of follow-up, 2,520 individuals developed dementia. The number of patients with dementia or cardiovascular risk factors increased slightly but steadily. Annually, the number of new cases of dementia increased from 0.5% in 2009 to 2.2% in 2017. Both Poisson regression and Cox’s proportional hazard model showed statistically significant relationships between hypertension, diabetes, dyslipidemia and obesity and dementia onset (p < 0.001), hazard ratio equals to 0.82, 1.11, 0.68 and 0.58, respectively. In other words, people with hypertension, dyslipidemia and obesity being managed in primary care are less likely to develop dementia. These findings support the hypothesis that good control over chronic diseases may benefit cognitive health.

